# Daily exposure to virtual nature reduces symptoms of anxiety in college students

**DOI:** 10.1038/s41598-023-28070-9

**Published:** 2023-01-23

**Authors:** Matthew H. E. M. Browning, Seunguk Shin, Gabrielle Drong, Olivia McAnirlin, Ryan J. Gagnon, Shyam Ranganathan, Kailan Sindelar, David Hoptman, Gregory N. Bratman, Shuai Yuan, Vishnunarayan Girishan Prabhu, Wendy Heller

**Affiliations:** 1 Virtual Reality and Nature Lab, Clemson University, Clemson, SC USA; 2 Department of Parks, Recreation and Tourism Management, Clemson University, Clemson, SC USA; 3 Department of Natural Resources and Environmental Sciences, University of Illinois at Urbana-Champaign, Urbana, IL USA; 4 College of Education, University of Illinois at Urbana-Champaign, Champaign, IL USA; 5 School of Mathematical and Statistical Sciences, Clemson University, Clemson, SC USA; 6 University of North Florida, Jacksonville, FL USA; 7 INVIROVR Inc., Santa Fe, NM USA; 8 School of Environmental and Forest Sciences, University of Washington, Seattle, WA USA; 9 Systems Engineering and Engineering Management, University of North Carolina at Charlotte, Charlotte, NC USA; 10 Department of Psychology, University of Illinois at Urbana-Champaign, Champaign, IL USA

**Keywords:** Psychology and behaviour, Anxiety, Depression

## Abstract

Exposure to natural environments offers an array of mental health benefits. Virtual reality provides simulated experiences of being in nature when outdoor access is limited. Previous studies on virtual nature have focused mainly on single "doses" of virtual nature. The effects of repeated exposure remain poorly understood. Motivated by this gap, we studied the influence of a daily virtual nature intervention on symptoms of anxiety, depression, and an underlying cause of poor mental health: rumination. Forty college students (58% non-Hispanic White, median age = 19) were recruited from two U.S. universities and randomly assigned to the intervention or control group. Over several weeks, anxious arousal (panic) and anxious apprehension (worry) decreased with virtual nature exposure. Participants identifying as women, past VR users, experienced with the outdoors, and engaged with the beauty in nature benefited particularly strongly from virtual nature. Virtual nature did not help symptoms of anhedonic depression or rumination. Further research is necessary to distinguish when and for whom virtual nature interventions impact mental health outcomes.

## Introduction

Exposure to natural environments can improve mental health. At least 17 systematic reviews have summarized the growing literature on this topic^1^. Across these reviews, hundreds of observational and experimental studies show that nature exposure can decrease symptoms of anxiety and depression, risk of cognitive decline (i.e., dementia), and stress while improving cognitive functioning and development, emotion regulation, mood, and psychological well-being. Some of the strongest associations between nature exposure and health are seen for anxiety and depression, with 33% and 37% less relative risk for people living in greener neighborhoods^2^.

Accessing outdoor natural environments is not possible for all people. Nearly two-thirds of U.S. citizens live in cities^3^, and these areas often lack adequate densities of well-maintained, safe, and accessible greenspaces^4^. People who live near "greenspaces" (vegetation-rich areas) may not visit for other reasons, like being unaware these places exist, facing discrimination, feeling unsafe in these spaces, and not prioritizing visitation^5^. Some physicians promote nature exposure by prescribing visits to parks to address mental health issues^6^. Still, these prescriptions require regular nature "outings" and are limited to those with the motivation, physical ability, financial means, and time to visit. Other populations face physical and mental constraints to accessing nature outdoors, such as some clinical populations and people living and working in isolated and confined environments (i.e., polar regions, submarines, cargo ships, and outer space)^7,8^.

While it is crucial to address barriers to access greenspace in real life, some of the benefits of nature contact have been observed in studies of virtual nature^9,10^. Virtual nature involves pictures, videos, or immersive media (i.e., virtual reality [VR] headsets) presenting audio–video or multisensory simulations of natural environments^11^. VR headsets are likely to have stronger associated health benefits than flat screens because of their high levels of immersion^12^. Audiovisual combinations promote greater psychological benefits than audio or visual sensory inputs alone^13^. Correspondingly, researchers are increasingly studying simulated nature in VR headsets rather than other media^11^.

Nearly 40 studies have investigated the short-term health outcomes of virtual nature in VR^14,15^. Observed outcomes include many of those associated with actual nature exposure but extend to pain management, disordered eating, phobias, post-traumatic stress, and cognitive rehabilitation^16^. The most commonly studied outcomes are mood, stress, and perceptions that an environment captures a viewer's attention without effort and restores cognitive capacities ("perceived restorativeness")^11^. These outcomes are likely selected due to the study design. Interventions with single exposures require surveys or psychological measures sensitive to short interventions. However, outcomes like clinical diagnoses or symptoms of anxiety and depression have strong societal implications owing to their growing global burden and treatment challenges^17,18^. Examining whether the therapeutic effects of nature on symptoms of anxiety and depression, as well as underlying causes (e.g., rumination), extend to VR is required to evaluate virtual nature’s clinical significance^19,20^.

Three prior studies of repeated exposures to virtual nature and anxiety or depression outcomes provide insights into its clinical significance. Veling et al. conducted a randomized control trial with 50 patients receiving treatment for mood disorders^21^. Comparisons were drawn between 360-degree videos of diverse natural landscapes (i.e., beaches, mountains, and underwater) and a guided meditation condition with audio tracks but no visual stimuli. The nature videos were created by the company Atmosphaeres and shown in a Samsung Gear VR headset using a Samsung Galaxy S6 smartphone (2560 × 1440 px, 60 Hz refresh rate). Both conditions were 10-min in duration and 10 days in length. The authors found a 10% reduction in depressive symptoms and a 29% reduction in anxiety symptoms for the virtual nature intervention. Depressive symptoms also decreased in the control condition. Differences between virtual nature and control conditions approached significance for anxiety but not depressive symptoms.

Lakhani et al. used a crossover design to study the effects of three 20-min viewings of natural landscapes and wildlife in 360-degree videos^22^. These were produced by the National Geographic and English broadcaster David Attenborough and shown in an Oculus Go headset (2560 × 1440 px, 60 Hz refresh rate). Data from 16 participants receiving spinal cord rehabilitation were collected. The control condition had no VR exposure. Participants were randomized to engage in VR or the control condition in the first vs. second week of the study. The results indicated reductions in depressive symptoms for both conditions. Only one subgroup of six patients showed significant reductions in symptoms throughout the VR intervention.

Reynolds et al. conducted a third crossover trial among 38 women with metastatic breast cancer. Participants saw one of two virtual nature experiences for an average of 13-min per day for one week^23^. These experiences included Ripple VR, which presented 360-degree videos of a beach, waterfall, and mountain from Mixt Studio, and Happy Place, a commercially available VR application with a highly stylized, animated camping scene developed by the company Hjärtat. Ripple VR allowed participants to write their names in the sand, stack rocks, and teleport between mountains and lakes. These experiences were presented in a Pico Goblin headset (1440 × 1280 px, 70 Hz refresh rate). No control condition was used. The authors found significant decreases in depressive and anxiety symptoms from the virtual nature intervention. Subsequent analyses reported these beneficial impacts were only observed for participants who did not feel strongly "connected" to nature at the beginning of the study^24^.

The current study was designed in response to the limited and conflicting results for repeated virtual nature exposure and mental health outcomes. We conducted a randomized control trial to test the impacts of daily exposure over at least a three-week period on college students' symptoms of anxiety, depression, and rumination. We chose this population since students may be particularly disposed to utilize nature exposure in VR settings, such as the "metaverse" (virtual environments that are interactive, immersive, and collaborative)^25^. Unlike past studies that examined depression or anxiety as unitary phenomena, we measured distinct dimensions of anhedonic depression, anxious arousal (panic), and anxious apprehension (worry), which differ in their psychological, physiological, and neurological characteristics^26–28^. These measures recognize the complex but precise symptoms of depression and anxiety. Somatic symptoms are relatively specific to anxiety, while low positive affect and anhedonia (lack of pleasure) are more specific to depression^29^. Anxiety can be further differentiated between anxious arousal, defined as physiological hyperarousal as well as feelings of panic and tension, and anxious apprehension, defined as chronic worry with verbal dwelling commonly on possible negative outcomes of future events^27^. Our daily intervention was limited to 4-min, which may elicit larger effects than longer durations^30^. Relatedly, we extended the study period over multiple weeks to evaluate longer-term exposures and outcomes. We included a control condition with no assigned intervention to determine the causal effects of exposure. We also controlled for behavioral changes across the study period that may impact mental health status. Finally, we considered measures related to nature connectedness that might moderate the effectiveness of virtual nature^23^. Our primary objective was to evaluate the impacts of daily virtual nature exposure on symptoms relative to no exposure.

## Results

### Sample characteristics

Table 1 shows the characteristics of the sample (N = 40). Additional descriptions of select variables (engagement with beauty and outdoor nature experiences) are provided in Table S1.

**Table 1:** Sample characteristics.

	Total (N=40)			Virtual Nature (N=24)			Control (N=16)		
*Female (N[%])*									
Female	30 (75%)			17 (70.8%)			13 (81.2%)		
Male	10 (25%)			7 (29.2%)			3 (18.8%)		
*Semester (N[%])*									
During COVID-19 pandemic	22 (55%)			14 (58.3%)			8 (50%)		
Not during COVID-19 pandemic	18 (45%)			10 (41.7%)			8 (50%)		
*VR experience (N[%])*									
No	12 (70%)			8 (33.3%)			4 (25%)		
Yes	28 (30%)			16 (66.7%)			12 (75%)		
*Baseline engagement with beauty (N[%])*									
Low (≤5.0 on 1=low, 7=high scale)	16 (40%)			10 (41.7%)			6 (37.5%)		
High (>5.0)	24 (60%)			14 (58.3%)			10 (62.5%)		
*Outdoor nature visits in past year (N[%])*									
Low (≤6-9 times)	25 (62.5%)			16 (66.7%)			9 (56.2%)		
High (≥10-14 times)	15 (37.5%)			8 (33.3%)			7 (43.8%)		
*Lifetime camping experiences (N[%])*									
Low (≤1 time)	31 (77.5%)			17 (70.8%)			14 (87.5%)		
High (>1 time)	9 (22.5%)			7 (29.2%)			2 (12.5%)		

	*Pre*	*Post*	*p*	*Pre*	*Post*	*p*	*Pre*	*Post*	*p*
Sleep (M[SD])	7.3(0.9)	6.8(1.5)	.085	7.4(1.0)	7.2(1.2)	.60	7.2(0.8)	6.3(1.7)	.050
Physical activity (M[SD])	47.4(53.8)	40.0(24.3)	.34	45.2(60.3)	34.7(19.5)	.37	50.6(43.8)	48.1(28.9)	.76
Worry (M[SD])	52.0(13.8)	50.2(15.1)	.13	52.0(16.4)	47.9(17.4)	.006	52.1(9.1)	53.5(10.7)	.49
Panic (M[SD])	25.5(7.2)	23.0(7.1)	.052	26.6(8.0)	22.6(6.6)	.012	23.7(5.7)	23.8(8.1)	.99
Depressive symptoms (M[SD])	50.8(13.0)	52.2(12.3)	.44	50.1(14.3)	50.8(12.6)	.77	52.0(11.0)	54.4(11.9)	.34
Rumination (M[SD])	37.9(7.7)	39.2(6.7)	.11	37.7(8.0)	39.1(7.2)	.15	52.1(9.1)	39.3(6.0)	.45

**Figure 1:**
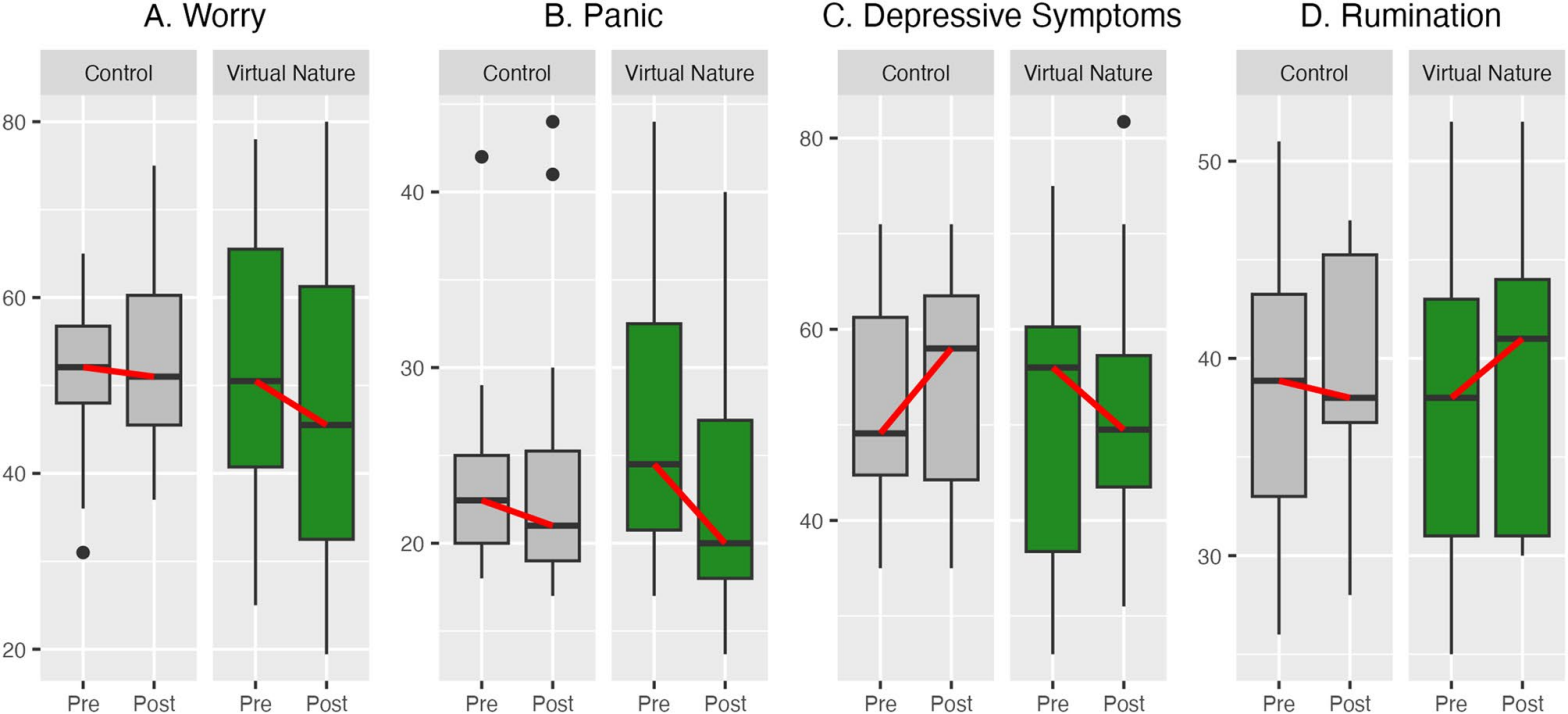
Impacts of virtual nature relative to a control condition (no intervention) on two dimensions of anxiety symptoms (worry (**A**) and panic (**B**)), depressive symptoms (**C**), and an underlying cause of poor mental health: rumination (**D**) (N = 40). Greater decreases correspond to stronger benefits of exposure for mental health. Red lines connect median values pre- and post-intervention. The time between the pre- and post-intervention was three weeks for the 18 students (45% of the total sample) who participated before the COVID-19 pandemic and four weeks for the 22 students (55% of the total sample) who participated during the COVID-19 pandemic.

Differences in outcome average pre/post values differ from Fig. 1, which shows medians in boxplots.

Three-quarters of the participants were female. Just over half of the participants completed the study during a COVID-19 pandemic semester (i.e., Fall 2022). Ages ranged from 18 to 22 years (M = 19.3, SD = 1.2). Participants identified as non-Hispanic (NH) White (N = 23, 57.5%), NH Asian (N = 11, 27.5%), NH Black (N = 3, 7.5%), NH multiracial (N = 1, 2.5%), or Hispanic (N = 2, 5%). On average, the 24 participants who completed the virtual nature condition adhered to the treatment 5.4 times/week (SD = 1.1, range = 2.7–7.0). Baseline comparisons for study outcomes were not different between conditions (worry, *p* = 0.98; panic, *p* = 0.19; depressive symptoms, *p* = 0.63; rumination, *p* = 0.84).

Data were available for an additional 37 students who chose not to participate. This sample was only a subset of the total number of invited eligible students (see Methods: Participants). A smaller share of these non-participants had experienced VR than participants, 43.2% vs. 70.0%, *X*^*2*^(1, N = 77) = 4.58, *p* = 0.032. Other comparisons did not show differences between these two groups, including outdoor nature visits and lifetime camping experiences, *ps* = 0.45 and 0.47, respectively.

### Effect of virtual nature on symptoms

Unadjusted values shown in boxplots suggested that virtual nature decreased worry, panic, and depressive symptoms while increasing rumination (Fig. 1A–D). In the control condition, depressive symptoms appeared to worsen while worry, panic, and rumination remained constant. Not all trends in mean comparisons were statistically significant, however. Decreases in worry and panic were significant for the virtual nature condition, *ps* = 0.006 and 0.012, respectively. No other changes in mental health approached significance for either condition, *p* > 0.10.

Adjusted models showed that decreases in worry with virtual nature exposure remained significant after accounting for gender, semester, and changes in sleep and physical activity (Table 2). Worry decreased by an average of 6.3 points relative to the control condition, *p* = 0.018. Given that this measure ranged from 16 (none of the survey items "being typical" of participants) to 80 (all items being "very typical"), the observed change corresponded to a 9.8% average decrease in worry.

**Table 2:** Regressing mental health changes on daily virtual nature exposure (N = 40).

	*Δ Worry*				*Δ Panic*				*Δ Depressive symptoms*				*Δ Rumination*			
	M1		M2		M1		M2		M1		M2		M1		M2	
	*β*	*p*	*β*	*p*	*β*	*p*	*β*	*p*	*β*	*p*	*β*	*p*	*β*	*p*	*β*	*p*
Female	− 0.07	0.70	− 0.14	0.39	0.01	0.93	− 0.05	0.78	0.19	0.26	0.19	0.28	0.21	0.22	0.22	0.21
COVID semester	0.01	0.97	0.13	0.52	0.14	0.53	0.24	0.27	− 0.19	0.36	− 0.19	0.40	0.10	0.64	0.08	0.72
Δ Sleep	− 0.07	0.73	− 0.03	0.87	− 0.07	0.74	− 0.04	0.86	0.02	0.92	0.02	0.92	0.20	0.33	0.20	0.35
Δ Physical activity	0.03	0.88	0.06	0.72	0.00	0.98	0.03	0.86	0.12	0.45	0.13	0.46	− 0.09	0.60	− 0.09	0.58
Virtual nature			− 0.41	0.018**			− 0.33	0.058^†^			− 0.02	0.93			0.07	0.69
R^2^/adj. R^2^	0.011/ − 0.10		0.16/0.041		0.036/ − 0.074		0.13/0.006		0.075/ − 0.031		0.075/ − 0.061		0.082/ − 0.023		0.087/ − 0.048	

M1 = Model 1, which provides a base model without the virtual nature intervention; M2 = Model 2, which adds the virtual nature intervention to the base model; **p < .01, ^†^*p* < .10.

Adjusted models also showed that decreases in panic with virtual nature exposure approached significance. Panic decreased by an average of 5.0 points relative to the control condition, *p* = 0.058.

No other significant changes in mental health were observed in the adjusted models. Changes in depressive symptoms and rumination relative to the control condition were non-significant. The final models predicted 16%, 13%, 8%, and 9% of the variance in worry, panic, depressive symptoms, and rumination changes, respectively.

**Figure 2:**
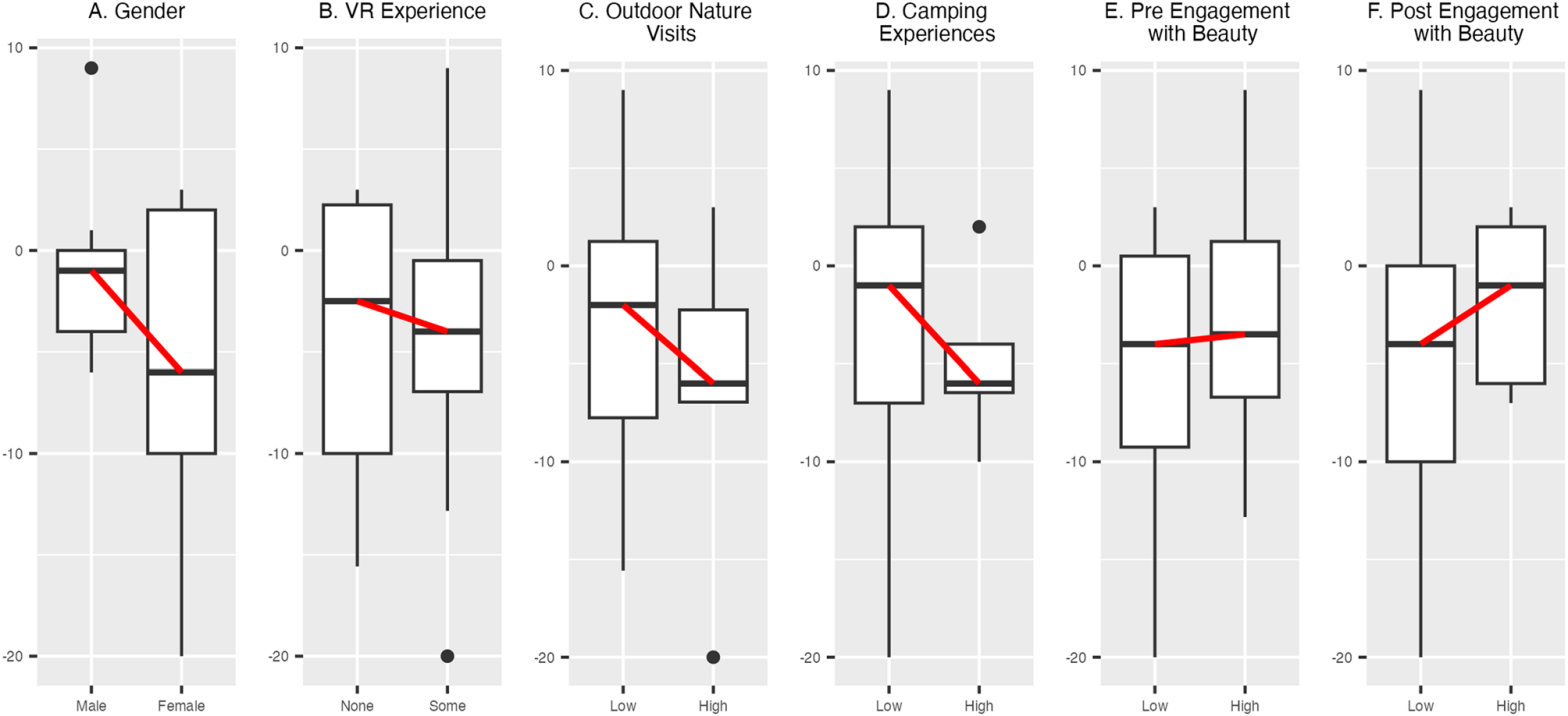
Moderating effects of gender (**A**), VR experience (**B**), exposure to outdoor nature (**C, D**), and engagement with beauty (**E, F**) for impacts of virtual nature on changes in worry (N = 23). Greater negative values correspond to stronger mental health exposure benefits. Red lines show changes in median values between conditions.

Gender, VR experience, and outdoor nature experience moderated the impact of virtual nature on changes in worry (Fig. 2A–D, Table S2). Female participants showed greater decreases than male participants, *p* = 0.0066. Participants with VR experience before the study also showed greater decreases than new VR users, *p* = 0.0070. In addition, participants with more visits to outdoor nature in the past year and lifetime camping experience showed greater decreases than participants with less outdoor nature exposure, *p*s = 0.0070 and 0.0066, respectively.

Higher levels of engagement with beauty also moderated the impact of virtual nature on changes in worry (Fig. 2E,F, Table S2). Participants with low baseline levels experienced greater decreases in worry than those with high baseline levels, *p* = 0.0089. Similarly, participants with low post-intervention levels experienced greater decreases in worry than participants with high post-intervention levels, *p* = 0.0074. Engagement with beauty values also changed over the study period. Levels decreased by 8.2% in the virtual nature condition and 9.4% in the control condition. Differences were significant only in the virtual nature condition, *ps* = 0.026 for virtual nature and 0.13 for control (Table S1).

## Discussion

Hundreds of studies document the benefits of actual or virtual exposure to nature on mental health^1,11^. The beneficial effects of virtual exposure are important for people with limited physical capacity or who face social and mental barriers to accessing safe outdoor environments. The expected impacts of virtual nature on mental health are largely drawn from short-term exposures and outcomes, such as stress, mood, and perceived restorativeness^11,15^.

We conducted one of the first longitudinal experiments of daily virtual nature exposure and clinical measures of mental health, namely symptoms of anxiety and depression and a potential cause of depression: rumination. Among our sample of 40 college students, anxious apprehension (worry) decreased with virtual nature exposure relative to no exposure. These changes remained significant after adjusting for gender, semester, and sleep and physical activity changes. Beneficial changes in anxious arousal (panic) were also observed and approached significance in adjusted models. No significant effects for depressive symptoms or rumination were observed.

Our findings are largely consistent with three prior studies that examined longitudinal exposure to virtual nature and clinical outcomes. Like the 10-day intervention by Veling et al., we found stronger effects of 360-degree nature videos on anxiety than on depressive symptoms^21^. The 3-day intervention by Lakhani et al. only measured depressive symptoms^22^. They did not find robust results for this outcome; only one of their experimental groups experienced drops over the intervention period. In contrast, the results of a 7-day intervention by Reynolds et al. differed from ours since they found decreases in anxiety and depression^23,24^. However, their study lacked a control condition to conclusively ascribe these changes to virtual nature in VR. Reynolds et al. also found clinically significant changes in depressive symptoms and not anxiety symptoms. The authors suggested these findings resulted from a "floor effect" whereby participants showed relatively low anxiety levels from the onset. The authors also noted the low internal reliability of their anxiety measure.

Research with singular exposures to virtual nature is largely silent on why daily virtual nature exposure might impact anxiety more than depressive symptoms. The findings of Chin et al. suggest one possible explanation^24^. Only women with weaker feelings of nature connectedness showed beneficial effects of virtual nature for depression. Our sample may have been dominated by college students with higher levels of connectedness and benefited little from our intervention. We measured engagement with beauty, which is conceptually and empirically distinct from connectedness to nature but still shows strong positive correlations with that measure^31^. Our participants' average engagement with beauty value was 5.1 on a 1 (low) to 7 (high) scale. If depressive symptoms only improve with virtual nature among people with low nature connectedness, our sample was unlikely to show effects. Meanwhile, virtual nature exposure may impact anxiety symptoms regardless of people’s nature connectedness levels.

Similarly, our findings for virtual nature not benefiting rumination are difficult to understand with existing research. Several studies show outdoor nature exposure can reduce this maladaptive pattern of self-referential thought and risk factor for depression^19,20,32^. We are unaware of studies on repeated virtual nature exposure and rumination. One study of a single dose of virtual nature found that state rumination decreased after viewing a slideshow of nature images; however, the same findings were found after viewing a slideshow of urban images without greenery^33^. This finding suggests that viewing a slideshow itself produced the desired impact regardless of the environments shown. Given the strong connection between depression and rumination^20^, it is possible for the effects of virtual nature on rumination to be moderated by connectedness to nature. Future research should test this hypothesis since the results may also help explain our findings for depressive symptoms.

We found that engagement with beauty changed across our study period. Significant decreases in the virtual nature condition and decreases that approached significance in the control condition were observed. Engagement with beauty and nature connectedness are expected to increase with nature contact and explain the downstream psychological benefits of exposure^31^. In past research, a single 10-min exposure to 360-degree videos of similar landscapes to those presented here (with the exception of rainforests) improved nature connectedness among participants with low baseline levels^34^. Investigations of the dose–response curves between concepts related to nature connectedness and repeated exposures to virtual nature are warranted. An ongoing systematic review that evaluates the capacity of virtual nature to increase nature connectedness may discover relevant findings^35^.

Another possible explanation for our divergent findings for anxiety and depressive symptoms relates to the mechanisms of action by which virtual nature might impact mental health. Virtual nature is likely to act through distraction and/or relaxation pathways^36^. The only study with repeated exposures that found consistent benefits for depressive symptoms included virtual nature experiences with interactions, like writing in the sand, moving virtual objects around, and teleporting between locations^23^. Our study and others that failed to find benefits of repeated exposure provided no interactive opportunities^21,22^. Interactions may distract participants from negative emotions by refocusing attention. Comparatively, non-interactive and interactive experiences may activate the parasympathetic nervous system through relaxation, which may alleviate anxiety symptoms.

We found that gender, VR use, and outdoor nature experience moderated the impacts of virtual nature on worry. Gendered results could be explained by women tending to benefit more than men from nature access due to biological differences, gender roles and norms, and differences in psychological relationships with nature^37^. Meanwhile, results for VR use and outdoor nature experience conflict with some past research on singular exposures to virtual nature^38^. The direction of these findings suggests that people familiar with virtual nature stimuli–both in terms of hardware and imagery–may benefit more than those who are less familiar.

Further research is needed to test these explanations. Participant's lived experiences and emotional connections with nature appear to affect their derived benefits of virtual nature^25^. Data on time in nature outdoors should be collected in studies of virtual nature to evaluate how time outdoors influences adherence and efficacy of time in VR. Researchers can track the number of minutes participants spend outdoors using mobile apps like NatureDose™ (NatureQuant LLC, Bend, OR) or calculate greenspace access using residential addresses and remotely sensed data^39^. Qualitative inquiry could help interpret differences in how participants emotionally respond to different types of natural landscapes–both real and virtual^40^. Non-interactive and interactive VR experiences could test the mechanisms underlying health benefits of outcome. Brain imaging studies could validate these findings and link them to environmental neuroscience^41^. Exposure to outdoor environments^20,42–45^ and virtual nature^44–49^ can elicit electrical activation and blood flow changes in brain regions associated with mental health. Distinguishing the clinical conditions studied here is available with functional magnetic resonance imaging (fMRI)^28^. Repeated brain scans would be necessary to validate our findings despite their financial and logistical challenges. Comparable studies of nature exposure and repeated brain imaging are rare^45^, but notable exceptions exist^20,43^. Importantly, stronger beneficial effects of nature exposure using mobile brain scanning approaches (i.e., functional near-infrared spectroscopy [fNIRS]) have been shown for outdoor settings than for videos^48^. This finding reinforces the need to control for outdoor exposure in virtual nature studies. Mental health or brain imaging changes from outdoor exposure may overwhelm the more subtle changes from virtual exposure.

The findings of this study reinforce a growing body of literature that finds virtual nature is an effective, safe, and acceptable intervention for mental health promotion^15^. While actual outdoor nature is likely to have stronger effects than virtual nature, virtual nature can produce an array of beneficial effects^10^. These extend beyond physiological, affective, and cognitive restoration to social, ecological, and "transcendent" (altered state of consciousness, or revelations where the self and environment are perceived anew) restoration^50^. Virtual nature should be offered to populations who do not have safe access to outdoor settings due to psychological, physical, residential, or other barriers. In particular, virtual nature could be recommended to clinical populations^16^ and people living and working in isolated and confined environments (i.e., polar regions, submarines, cargo ships, and outer space)^7,8^.

Virtual nature interventions should minimize the potential for adverse effects. Regarding cybersickness, potential participants could be screened using a validated survey battery for motion sickness (i.e., Reason and Brand Motion Sickness Susceptibility Questionnaire [MSSQ]^51^; Motion History Questionnaire [MHQ]^52^). Baseline levels of the Simulator Sickness Questionnaire (SSQ)^53^ have also been used for this purpose^54,55^. We are unaware of virtual nature studies using these susceptibility measures despite their use in the broader VR literature and correlations with cybersickness^56^. Testing susceptibility may be particularly important among populations vulnerable to cybersickness, such as women, people possessing a neurological disorder or phobia, and people with limited experience using technology^57^. Techniques to minimize cybersickness in the creation of virtual nature interventions also exist. When using a 360-degree camera, videos can be captured exclusively on a stationary tripod, as we have done here, or, when movement is desired, videos can be stabilized using an electronic handheld gimbal for moving video^58^ with the horizon clearly visible^59^. Aside from cybersickness, HMDs can induce visual fatigue, also called eyestrain and describing the physiological strain or stress resulting from excessive demands on the visual system^60,61^. Techniques to reduce visual fatigue may include presenting monoscopic videos as opposed to stereoscopic videos (different viewing angles presented to each eye for depth perception); limiting durations of HMD use to 20 min.; and avoiding VR after extended use of other devices that emit blue light (i.e., phones, computers)^61^. With the increasing quality of HMDs and 360-degree camera technology, cybersickness may play a decreasing role in virtual nature counterindications. Meanwhile, visual fatigue may play an increasing role in who should be provided virtual nature interventions, given many populations’ increasingly high levels of screen time^62,63^.

Our study has strengths and limitations. We differentiated between dimensions of anxiety and depressive symptoms using scales with high levels of discriminant validity. We included a control condition to account for student mental health changes over the semester. However, our choice of the control condition could be viewed as a weakness since we could not account for the potential "novelty" effect of VR, whereby newer users may respond more favorably to VR experiences^64^. We expect such effects to be minimized by the 15 or more exposures our VR group experienced during the intervention. Our sample consisted of healthy undergraduate students across multiple calendar seasons. While results derived from this population may not generalize to broader populations, college students suffer from mental health issues at high rates, and their risk of anxiety and depression heightened during the COVID-19 pandemic^65^. Students who participated during the pandemic may have been particularly sensitive to changes in mental health from our intervention. Still, the confounding effects of conducting a study over three semesters with multiple calendar seasons may have influenced our results^66^. Last, we did not test for the possibility of negative emotional responses, such as boredom or fear, from virtual nature exposure. Such findings have been reported in studies among young adults^40^, including when nature videos are presented without nature sounds^47^, and among dementia patients^67^. Individual responses to certain landscapes presented in HMDs could explain the modest effect sizes for anxiety symptoms and null results for depressive symptoms. This possibility warrants future investigations that measure landscape preferences and lived experiences alongside mental health outcomes.

In conclusion, we found evidence that daily doses of virtual nature exposure can decrease anxiety for college students. More research is needed to distinguish the mechanisms by which exposure impacts mental health to identify when and for whom it benefits clinical outcomes.

## Methods

### Participants

We recruited healthy young adults from two large public universities in the U.S. using a convenience sampling approach. Undergraduate students from the University of Illinois at Urbana-Champaign (UIUC), Urbana, IL, were recruited in the fall of 2019 and early spring of 2020 (before mid-March) semesters while undergraduate students from Clemson University, Clemson, SC were recruited in the fall 2020 semester. A recruitment message to a screening survey was distributed to the UIUC Department of Psychology Subject Pool and an online undergraduate course with 700 enrolled students. At Clemson University, a recruitment message was distributed to an in-person class with approximately 900 enrolled students. Courses at Clemson and UIUC met general education requirements; therefore, students represented a diverse set of academic majors. Students completed a screening survey and were eligible if they were between 18 and 29 years old, did not take daily psychoactive medications, had normal or correct-to-normal vision and hearing, and did not have astigmatism of more than one diopter between their eyes. The study was approved by the UIUC Institutional Review Board (Protocol #19401) with a reciprocal agreement at the Clemson University Institutional Review Board and carried out in accordance with the current version of the Declaration of Helsinki. Informed consent was obtained from all participants.

Sixty students (26 from UIUC and 34 from Clemson) started the study. One UIUC student did not watch videos in the final week and was excluded. Another UIUC student reported 23 h of sleep over the last week and was excluded. One Clemson participant completed both surveys but provided too few responses for at least one survey battery to impute all values. A complete dataset for the screening survey was unavailable when data analysis occurred. Missing data resulted from personnel changes, institutional affiliation changes, and software administrative access limitations. Relatedly, potential errors in participants’ unique identifiers in the screening survey data resulted in unmatched observations between these data and the pre- and post-intervention survey data. Data from 40 participants (18 from UIUC, 22 from Clemson) were ultimately available for analysis.

### Procedure

Participants were randomly assigned to an intervention or control condition. Both conditions completed online surveys on VR use and pre- and post-surveys on mental health outcomes. The duration of the study depended on the university. Our original schedule was a four-week intervention period, and we used this period for the two semesters at UIUC. Restrictions on participants' capacity to engage in research during the COVID-19 pandemic required that we shorten the study period at Clemson to three-weeks.

**Figure 3:**
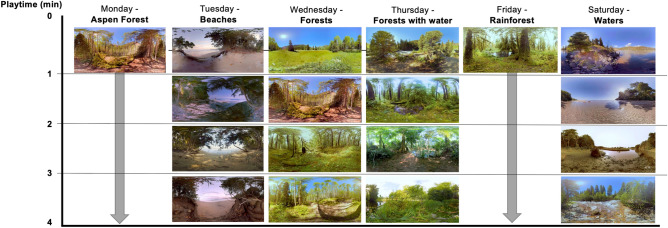
360-degree videos shown in the virtual nature intervention (Courtesy of INVIROVR).

Students in the intervention condition were loaned VR headsets for the study. Headsets were provided in person before the pandemic and mailed with a prepaid return shipping label during the pandemic to avoid in-person contact. Students were asked to watch one of six 360-degree videos on Monday through Saturday of each week (Fig. 3). The videos were 4-min in length. The control condition received no intervention or instructions beyond completing the online surveys.

### VR content

The 360-degree videos were created by the company INVIROVR (https://invirovr.com/) using a GoPro Omni camera rig (GoPro, Inc., San Mateo, CA, USA). This frame holds six GoPro Hero4 Black cameras with fisheye lenses facing different directions. The frame was fixed on a tripod at eye level (2-m) to simulate an on-site experience. Videos were recorded during sunny or partly cloudy weather. The individual videos were stitched together to create spherical 360-degree videos.

A pilot study tested 58 possible natural landscapes with nine UIUC graduate students and faculty members familiar with VR research. We defined “natural landscapes” as environments abundant in features (i.e., plants, water, rocks, and minerals) with little or no apparent evidence of human presence or intervention, in alignment with previous reviews on the health benefits of nature exposure^68,69^. These participants watched 360-degree videos created by INVIROVR, including forests, beaches, deserts, lakes, grasslands, and alpine areas. The videos varied by duration, type, and number of transitions. Data from focus groups revealed three conclusions. First, 4-min durations were long enough to be perceived as restorative but not too long to induce boredom. Second, beaches and water bodies were preferred over forested or other green landscapes, but these types were preferred over rocky landscapes. Third, videos with transitions were preferred over videos without transitions.

A subsequent study with 95 participants was conducted with six 4-min 360-degree videos using a subsample of the above landscapes^40^. The locations spanned from Costa Rica, the northern United States (i.e., Upper Peninsula of Michigan), to the Rocky Mountain West (i.e., Colorado and New Mexico) and constituted a mixture of the three major components of natural landscapes–plants, water, and rocks/minerals–with high amounts of plant biomass, which has been shown to increase the beneficial effects of VR exposure^68,70^. Most videos included four scenes presented for 1-min each with a 3-s crossfade between scenes. Since our pilot data were limited in generalizability, we included two videos with a single scene shown for the entire 4-min. A qualitative investigation found some scenes were preferred more frequently than others but at least one participant "favorited" each of the six videos^40^. Therefore, we elected to use all six videos (Fig. 3). Locations were expected to be unfamiliar to participants so restorative effects were similar across the sample^71^. Videos were selected to exclude cars, traffic noises, buildings, airplane flyovers, jet trails, or people since these elements can affect preferences and responses to natural landscapes^11,40,72^. After our study began, we observed that one video included a 16-s segment of three people barely visible in the background.

Videos were presented in Oculus Go headsets (2560 × 1440 px, 60 Hz refresh rate). The sound was played in on-ear headphones. Headsets, controllers, and headphones were cleaned between participants according to the manufacturer and best practice guidelines with disinfecting wipes and an ultraviolet (UV) box (CleanBox, Nashville, TN, USA)^73^.

### Measures

We measured anxious apprehension (elsewhere labeled as "worry") with the Penn State Worry Questionnaire^74^. This instrument asks respondents 16 items about their tendencies to worry, inability to control these tendencies, and resulting negative impacts on a 6-point scale (1 = not at all typical of me, 6 = very typical of me). The scale has shown high internal consistency in past research and our sample (Cronbach's alpha = 0.93)^27,74^.

We measured anxious arousal ("panic") with items from the Mood and Anxiety Symptom Questionnaire (MASQ)^75,76^. These items show high levels of discriminant validity from other mood disorders and internal consistency, including in our sample (Cronbach's alpha = 0.85)^27^. Participants were asked to rate their symptoms over the past week on a 5-point scale (1 = not at all, 5 = extremely). Examples included being startled easily, trembling or shaking, muscle twitches or trembles, and feeling dizzy or lightheaded. The UIUC Institutional Review Board required one item asking about suicidal thoughts removed. Therefore, data collection at that institution had 16 items for this index, while data collection at Clemson had 17 items.

Another 22 items from the MASQ were used to measure depressive symptoms^27^. The internal consistency in past research and our sample was high (Cronbach's alpha = 0.91)^27^. Participants were asked to rate symptomology over the past week on the same 5-point scale (1 = not at all, 5 = extremely). Example items included feeling withdrawn from other people, nothing is very enjoyable and cheerful or really happy (reverse coded).

A principal driver of depression and outcome shown to benefit from nature exposure was also measured: rumination^20,77^. Rumination is the maladaptive pattern of self-referential thought^78^. The concept was measured with 12 items from the Rumination Reflection Questionnaire (RRQ) on a 5-point scale (1 = strongly disagree, 5 = strongly agree)^79^, which showed high internal consistency in our sample (Cronbach’s alpha = 0.77). Example items included "it is easy for me to put unwanted thoughts out of my mind" and "I often find myself re-evaluating something I've done" (reverse coded).

Additional variables were measured to understand the sample characteristics and control for potential confounding effects. Gender and semester of participation were collected before the study began. The number of viewings per week among participants was retrieved from weekly online surveys by asking participants how many videos during the past week were watched to completion. Sleep quantity was measured with a single item: "how much sleep did you get on average during the past week?" Physical activity was measured with the Godin-Shephard Leisure-Time Physical Activity Questionnaire^80^. This consists of three questions about the frequency of light, moderate, and strenuous exercise in the past week. A summative score was calculated by summing the number of strenuous exercise bouts multiplied by nine, number of moderate exercise bouts multiplied by five, and number of light exercise bouts multiplied by three. The resulting scale has arbitrary units. More physically fit people tend to have ranges around 150 to 200, while less fit people tend to range from 50 to 100^80^.

Potential moderators were selected based on the results available during this study's conception^38,64^. The extent to which participants perceive themselves as emotionally and physiologically responding to beauty in the natural world was measured with the four-item subscale of the Engagement with Beauty Scale^81^. Items (i.e., "I notice beauty in one or more aspects of nature") were measured on a 7-point scale (1 = very unlike me, 7 = very like me). Scores were analyzed at baseline and the end of the intervention (week 4 for UIUC participants, week 3 for Clemson participants). The internal consistency was high (Cronbach's alpha = 0.80).

Baseline experiences outdoors in nature were captured with nature visits over the past year and camping experiences over the lifetime. The first item asked how often participants visited a "nature-based park" and provided local examples of city or county parks, forest preserves, botanical gardens, and private woodlands. Nine response categories were possible (1 = no times in the past year, 9 = five or more times per week for most weeks in the past year)^82^. No participants reported the highest response category. The second item asked participants how often they went camping overnight "in the wilderness." Five response options were provided (1 = no times in my life, 5 = more than ten times in my life). A complete listing of response options is available in Table S1.

We measured past VR use to capture the potential "novelty effects" of using a headset before the study^64^. To do so, we asked participants whether they had ever experienced virtual reality. Response options included yes and no as well as "not sure." No participants indicated they were unsure whether they had used VR.

### Analyses

Survey data were first screened for missing values. Five missing cases were found for rumination in the control condition and imputed using expectation maximization (EM)^83^. The single item measuring suicidal ideation was also imputed for the UIUC participants. The Clemson data were screened for systematic causes of missingness utilizing Little's test of missing completely at random (MCAR)^84^. Non-significant results indicated that data were MCAR (χ^2^ = 500.56, DF = 610, *p* > 0.05). An EM technique was employed to impute these data. Rates of missingness ranged from 25 to 28% for mental health outcomes and 49% for sleep and physical activity.

Sample characteristics were described based on the variable type. Continuous measures were reported with means and standard deviations or medians and interquartile ranges (IQRs) depending on distribution. Binary measures were reported as counts and frequencies. Differences between pre- and post-interventions were compared with paired sample *t-*tests.

We used four sets of stepwise regressions with changes in worry, panic, depressive symptoms, and rumination as the outcomes. Base models included gender, semester (during the COVID-19 pandemic or not), and sleep and physical activity changes. Change scores for time-variant confounders were used to simplify the models and optimize statistical power. The final models added the experimental condition as a dummy variable with the control as the reference group. Statistical significance was defined by *p* < 0.05. Analyses were completed in R V4.1.2 (R Foundation, Vienna, Austria).

Moderation tests were performed among the subsample of virtual nature participants. We compared the effects of virtual nature among women vs. men, past VR users vs. new VR users, and low vs. high levels of engagement with beauty, nature visitation, and camping experiences. Participants were categorized between the three latter variables into low vs. high levels using median splits: low engagement with beauty ≤ 5; low nature visits ≤ 4 (6 to 9 times in the past year); low camping experiences ≤ 2 (1 time over the lifetime).

## Supplementary Information

Supplementary Information.

## Data Availability

The datasets generated and analyzed during the current study and the scripts used to generate the results are available on the Open Science Framework (OSF) at https://osf.io/r73x5/ (https://doi.org/10.17605/OSF.IO/R73X5).
