# Supplementary material for: Daily exposure to virtual nature reduces symptoms of anxiety in college students
Source: Sci Rep. 2023 Jan 23;13:1239. doi: 10.1038/s41598-023-28070-9 (PMC9868517; doi:10.1038/s41598-023-28070-9)
Supplement: Supplementary file 1 — Supplementary Information. [file 41598_2023_28070_MOESM1_ESM.docx]

**Supplemental material**

**Daily Exposure to Virtual Nature Reduces Symptoms of Anxiety in College Students**

Matthew H. E. M. Browning^1,2*^, Seunguk Shin^3^, Gabrielle Drong^4^, Olivia McAnirlin^1,2^, Ryan J. Gagnon^2^, Shyam Ranganathan^5^, Kailan Sindelar^6^, David Hoptman^7^, Gregory N. Bratman^8^, Shuai Yuan^1,2^, Vishnunarayan Girishan Prabhu^1,9^, Wendy Heller^10^

^1^ Virtual Reality and Nature Lab, Clemson University, Clemson, South Carolina, USA

^2^ Department of Parks, Recreation and Tourism Management, Clemson University, Clemson, SC, USA

^3^ Department of Natural Resources and Environmental Sciences, University of Illinois at Urbana-Champaign, Urbana, IL USA

^4^ College of Education, University of Illinois at Urbana-Champaign, Champaign, IL USA

^5^ School of Mathematical and Statistical Sciences, Clemson University, Clemson, SC USA

^6^ University of North Florida, Jacksonville, FL USA

^7^ INVIROVR Inc., Santa Fe, NM USA

^8^ School of Environmental and Forest Sciences, University of Washington, Seattle, WA USA

^9^ Systems Engineering and Engineering Management, University of North Carolina at Charlotte, Charlotte, NC USA

^10^ Department of Psychology, University of Illinois at Urbana-Champaign, Champaign, IL USA

^*^ Corresponding author: [mhb2@clemson.edu](mailto:mhb2@clemson.edu)

**Table S1.** Detailed sample characteristics for moderating variables.

|  | **Total (N=40)** | | | **Virtual Nature (N=24)** | | | **Control (N=16)** | | |
| --- | --- | --- | --- | --- | --- | --- | --- | --- | --- |
| **Outdoor nature visits in the past year** |  | | |  | | |  | | |
| 0 times (1) | 3 (8%) | | | 1 (4%) | | | 2 (13%) | | |
| 1 time (2) | 3 (8%) | | | 2 (8%) | | | 1 (6%) | | |
| 2-5 times (3) | 14 (35%) | | | 8 (33%) | | | 6 (38%) | | |
| 6-9 times (4) | 5 (13%) | | | 5 (21%) | | | 0 (0%) | | |
| 10-14 times (5) | 7 (18%) | | | 3 (13%) | | | 4 (25%) | | |
| 2 times/month, most months (6) | 3 (8%) | | | 1 (4%) | | | 2 (13%) | | |
| 1 time/week, most weeks (7) | 3 (8%) | | | 3 (13%) | | | 0 (0%) | | |
| 2-4 time/week, most weeks (8) | 2 (5%) | | | 1 (4%) | | | 1 (6%) | | |
| **Lifetime camping experiences** |  | | |  | | |  | | |
| 0 times (1) | 13 (33%) | | | 5 (21%) | | | 8 (50%) | | |
| 1-3 times (2) | 18 (45%) | | | 12 (50%) | | | 6 (38%) | | |
| 4-6 times (3) | 2 (5%) | | | 2 (8%) | | | 0 (0%) | | |
| 7-10 times (4) | 1 (3%) | | | 1 (4%) | | | 0 (0%) | | |
| >10 times (5) | 6 (15%) | | | 4 (17%) | | | 2 (13%) | | |
|  | ***Pre*** | ***Post*** | ***p*** | ***Pre*** | ***Post*** | ***p*** | ***Pre*** | ***Post*** | ***p*** |
| **Engagement with beauty** (M[SD]) | 5.3(1.0) | 4.9(1.2) | .008 | 5.4(1.1) | 5.0(1.1) | .03 | 5.2(0.8) | 4.7(1.2) | .13 |
|  |  |  |  |  |  |  |  |  |  |

Notes: results of *t*-tests shown for engagement with beauty between pre- and post-intervention scores.

**Table S2.** Detailed effect modification results by gender, VR experience, exposure to outdoor nature, and engagement with beauty for impacts of virtual nature on changes in worry (N=24).

|  |  | **Med(IQR)** | **p** |
| --- | --- | --- | --- |
| **Gender** | | | .0066 |
|  | Male | -1(4) |  |
|  | Female | -6(12) |  |
| **VR experience** | | | .0070 |
|  | No | -2.5(12.2) |  |
|  | Yes | -4(6.45) |  |
| **Outdoor nature visits in the past year** | | | .0070 |
|  | Low | -2(9) |  |
|  | High | -6(4.7) |  |
| **Lifetime camping experiences** | | | .0066 |
|  | Low | -1(9) |  |
|  | High | -6(2.46) |  |
| **Baseline engagement with beauty** | | | .0089 |
|  | Low | -4(9.75) |  |
|  | High | -3.5(7.95) |  |
| **Post-intervention engagement with beauty** | | | .0074 |
|  | Low | -4(10) |  |
|  | High | -1(8) |  |

Notes: results of Wilcoxon rank sum tests shown, medians presented due to skewed variable distributions
